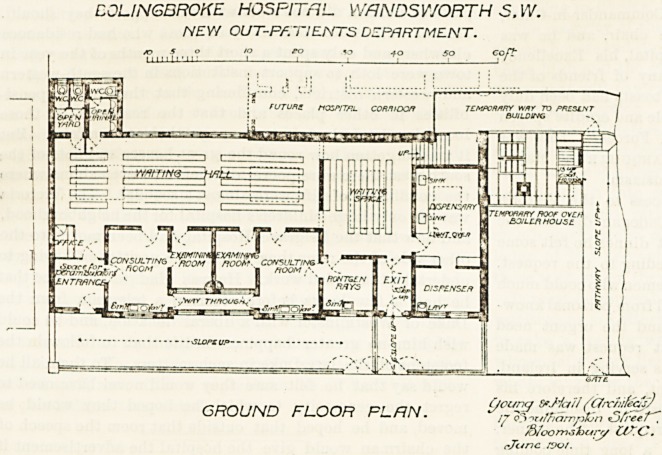# The Bolingbroke Hospital, Wandsworth

**Published:** 1901-11-30

**Authors:** 


### The Institutional Workshop.

### THE BOLINGBROKE HOSPITAL, WANDSWORTH.

NEW OUT-PATIENTS' DEPARTMENT.

As the present hospital was converted out of an old mansion, it will easily be understood that it is not suitable for its purpose, and a new scheme has been prepared which, when completed, will provide accommodation for 135 patients. The new scheme will comprise the hospital proper, administrative offices, nurses' quarters, and an out-patients' department. The latter is now being carried out. It is connected with the old hospital by means of a temporary passage, and it will only communicate with the new building by a door opening into the main corridor.

It is a one-story building consisting of a general

BOLINGBROKE HOSPITAL WANDSWORTH S.W.
NEW OUT-PATIENTS DEPARTMENT.

GROUND FLOOR PLAN.

Young & Hall (Architects) 17 Southampton Street, Bloomsbury W.C. June 1901.

waiting-room, two consulting rooms with an examining room to each, a waiting room close to the dispensary, and a room for Roentgen rays. These are arranged within a parallelogram of about 87 feet by 35 feet. The patients' entrance is at the extreme north-east corner of the parallelogram. Here also are the office and a place for perambulators. Passing the office the general waiting-room is entered, and opposite is the sanitary block, the closets being approached through a small open yard. The consulting-rooms, with their adjuncts, are placed alongside the general waiting-room. From the latter two doors open into the dispensary waiting-room. One of these doors leads to the temporary corridor, and the other to the exit. The arrangements are all very good, and the space has been carefully utilised. The whole of the internal walls up to 5 feet are lined with salt-glazed bricks, and the upper part with opalite. The floors are laid with Rust's mosaic. The contractors are Messrs. Turtle and Appleton, of Wandsworth, and the architects are Messrs. Young and Hall, of Southampton Street, W.C. The cost is not stated.

## Figures and Tables

**Figure f1:**